# Understanding the Role of Loneliness in the Relationships Between Post-Traumatic Stress Symptoms and Both Anxiety and Depressive Symptoms Among University Students: A Mediation Analysis

**DOI:** 10.3390/brainsci15080787

**Published:** 2025-07-24

**Authors:** Ilaria Riboldi, Cristina Crocamo, Chiara Alessandra Capogrosso, Francesco Bartoli, Jo Armes, Cath Taylor, Giuseppe Carrà

**Affiliations:** 1 School of Medicine and Surgery, University of Milano-Bicocca, Via Cadore 48, 20900 Monza, Italy; cristina.crocamo@unimib.it (C.C.); c.capogrosso@campus.unimib.it (C.A.C.); francesco.bartoli@unimib.it (F.B.); giuseppe.carra@unimib.it (G.C.); 2 Faculty of Health and Medical Sciences, School of Health and Sciences, University of Surrey, Stag Hill, Guildford GU2 7XH, UK; jo.armes@surrey.ac.uk (J.A.); cath.taylor@surrey.ac.uk (C.T.)

**Keywords:** loneliness, mental health, post-traumatic stress, university students

## Abstract

**Background/Objectives**: Both traumatic and stressful events, including major life changes, may contribute to post-traumatic stress symptoms (PTS), often associated with anxiety and depression. Feelings of loneliness may influence these relationships, whilst social support seems to mitigate the effects of stressful events on mental health. Our study thus aimed to evaluate the mediating role of loneliness in the relationships between PTS and both anxiety and depressive symptoms among university students. **Methods**: The data were from the CAMPUS study (0058642/21; FHMS 20-21 157), a survey on university students’ mental health in Italy and the UK. Using a logit model, mediation analyses were carried out to test whether the relationships between PTS and both anxiety and depressive symptoms might be mediated by loneliness. A path analysis was then performed to jointly test the associations between the Impact of Event Scale—Revised (IES-R)’s subscales and clinical domains. **Results**: Positive associations were found between PTS and both anxiety (*p* < 0.001) and depressive symptoms (*p* < 0.001). However, loneliness mediated approximately 22% of the effect of the PTS on anxiety symptoms (indirect effect: 1.04, 95% CI: 0.59; 1.48, *p* < 0.001) and approximately 33% of the effect of the PTS on depressive symptoms (indirect effect: 1.81, 95% CI: 1.22; 2.39, *p* < 0.001). Furthermore, the path analysis indicated associations between the IES-R’s hyperarousal subscale and both anxiety (coeff.: 0.34, *p* < 0.001) and depressive symptoms (coeff.: 0.27, *p* < 0.001). **Conclusions**: Along with the associations between PTS and both anxiety and depressive symptoms, our findings highlight the key role of loneliness in both these associations. Targeted interventions to reduce loneliness, especially for students exposed to traumatic events, may ultimately improve their mental health.

## 1. Introduction

According to the *Diagnostic and Statistical Manual of Mental Disorders, 5th Edition* (DSM-5), psychological trauma refers to the emotional and cognitive impacts of experiencing or witnessing events perceived as life-threatening, overwhelming, or deeply distressing [1]. However, stressful life events, though not necessarily life-threatening, can also significantly disrupt emotional well-being and daily functioning, reducing coping strategies and contributing to the development of post-traumatic stress symptoms (PTS) [2,3,4,5]. These events may include major life changes, such as the transition to university, which is, in particular, recognized as a period of increased psychological vulnerability, thus making students a relevant population for further investigation into PTS [6,7]. Indeed, the university experience, often framed as a time of opportunity and personal growth, may heighten emotional distress due to academic pressure, social isolation, and the challenges of adapting to a new environment [6,7]. According to the existing literature, PTS, characterized by intrusive thoughts, hyperarousal, emotional numbness, and avoidance behaviors, are closely linked to anxiety and depression, potentially exacerbating students’ psychological burden [8,9], whose symptoms are increasingly prevalent among university students [10].

However, emerging research emphasizes the key role of social support in buffering the effects of overwhelming stress and trauma [11,12]. Conversely, feelings of loneliness, particularly common among students who often face geographical relocation and cultural adjustment, can intensify vulnerability to stressful events and associated mental health issues [13,14,15]. Notably, loneliness has been shown to have bidirectional relationships with both depression and anxiety: while it increases the risk and severity of depressive and anxiety symptoms, it is also reinforced by them [16,17,18]. Although PTS have been consistently associated with both anxiety and depression, the potential mediating role of loneliness in these relationships has not been fully explored yet [8,9]. In particular, it can be argued that loneliness could partially mediate the relationship between PTS and students’ mental health and might represent potential targets for intervention.

Thus, benefiting from the cross-sectional data of the CAMPUS study, which used a large and representative sample of university students from Italy and the UK, we tested the hypotheses that (i) PTS and loneliness would be associated with anxiety and depressive symptoms and that (ii) the relationships between PTS and both anxiety and depressive symptoms would be at least partially mediated by loneliness.

## 2. Materials and Methods

### 2.1. Study Design and Setting

This study followed the Strengthening the Reporting of Observational Studies in Epidemiology (STROBE) checklist [19]. The data were from the second wave of the “Caring for and Assessing the Mental Health of Student Populations at Unimib and uniSurrey” (CAMPUS) study, a large survey, longitudinally assessing the mental health of university students enrolled at the University of Milano–Bicocca (Italy, Unimib) and the University of Surrey (the UK, UoS). An online survey was delivered by e-mail to students between October and November 2022. All the procedures were approved by the ethics committees of the University of Milano–Bicocca (registration number: 0058642/21) and the University of Surrey (registration number: FHMS 20-21 157). Students older than 18 provided their informed consent online. They could complete the survey in a private setting and quit at any time. The online platform (Qualtrics) anonymously tracked both complete and partial responses.

### 2.2. Measures

An extensive battery of instruments was used to gather information on both individual characteristics and psychometric measures, though only those relevant to this study are presented herein.

#### 2.2.1. Participant Information

We collected information on age, gender, living conditions, employment, and relationship status. Data on the degree course, on the years of study completed, as well as on the international background were also gathered. Academic performance was investigated using a self-reported binary indicator, e.g., “to be on track with exams”, compared with “not being on track”, while social connectedness was explored through the frequency of interactions, that is, daily/weekly versus monthly or less frequent interactions with relatives, friends, and classmates.

#### 2.2.2. Psychometric Measures 

Self-reported psychometric measures included the 7-item General Anxiety Disorder scale (GAD-7), the 9-item Patient Health Questionnaire (PHQ-9), and the 22-item Impact of Event Scale—Revised (IES-R). Both the English- and Italian-validated versions were used.

The 7-item General Anxiety Disorder scale (GAD-7) was used to assess symptoms of generalized anxiety experienced in the previous two weeks. Response options included “never”, “some days”, “more than half the days”, and “almost every day”, with scores of 0, 1, 2, and 3, respectively, and the relevant total score ranging from 0 to 21, with cutoff scores as follows: 0–4, minimal; 5–9, mild; 10–14, moderate; and 15–21, severe anxiety [20].

As far as the depressive symptoms’ assessment, we used the 9-item Patient Health Questionnaire (PHQ-9) [21], the self-administered version of the Primary Care Evaluation of Mental Disorders (PRIME-MD) [22]. The PHQ-9 enables screening, diagnosis, and severity assessment through nine items that reflect the symptoms of major depression, according to the DSM-IV, over the previous two weeks. Possible answers included “never”, “several days”, “more than half the days”, and “nearly every day”, and each item was rated from 0 (“never”) to 3 (“nearly every day”). The total score ranged from 0 to 27, according to these cutoff scores: 5–9, mild; 10–14, moderate; 15–19, moderately severe; and ≥20, severe depressive symptoms [21].

The 22-item Impact of Event Scale—Revised (IES-R) was used to assess symptoms of post-traumatic stress experienced in response to specific events. Response options included “not at all”, “a little bit”, “moderately”, “quite a bit”, and “extremely”, with scores of 0, 1, 2, 3, and 4, respectively. The total score ranged from 0 to 88 and reflected the overall symptom severity across three subscales: intrusion, avoidance, and hyperarousal. Although not intended as a standalone diagnostic tool for post-traumatic stress disorder (PTSD), a cutoff score of 33 or above is considered as indicative of a probable PTSD diagnosis, with higher scores suggesting the presence of PTS and increased psychological distress [23]. Finally, the participants were provided with an open-ended question to report and describe the specific life event they perceived as the most stressful. This approach offered qualitative insight into the nature of these experiences and their potential correspondence with DSM-5 Criterion A for PTSD. 

#### 2.2.3. Loneliness

Perceived loneliness was assessed through the UCLA Loneliness Scale (Version 3), which consisted of 20 items, each rated on a 4-point Likert scale, covering various aspects of social and emotional loneliness, according to these cutoff scores: 20–34, low; 35–49, moderate; 50–64, moderately high; and 65–80, a high degree of loneliness [24].

### 2.3. Statistics

After conducting validity and consistency checks on the collected data, we performed descriptive analyses to summarize the characteristics of the survey participants. We also addressed missing data, confirming that information on anxiety symptoms (assessed using the GAD-7, the first tool in the survey) was unavailable for early non-completers, defined as respondents with more than 30% unanswered items. We compared students who completed the survey with early non-completers to identify characteristics that might explain differences in the likelihood of the survey completion. Assuming that non-response was, at most, dependent on observed covariates, we excluded the possibility of data being missing not at random (MNAR). Descriptive statistics were reported for both continuous and categorical variables, stratified by the IES-R cutoff score. Potential differences in PTS were, thus, assessed across individual characteristics by running both chi-squared and Fisher’s exact tests for categorical variables, while Student’s T or Mann–Whitney U tests (consistently with data distribution) were run for continuous variables. In addition, Spearman’s rank correlation (ρ) was calculated to describe the associations between specific domains. Then, using a binary logistic (logit) model [25], we carried out mediation analyses to test whether the relationships between PTS (as an independent variable) and both anxiety and depressive symptoms (as dependent variables) were direct or whether a putative mediator variable (e.g., loneliness, as assessed using the UCLA scale) would account for the relationships between them. The putative mediator was entered in separate models to assess its individual impacts on the two distinct relationships, also considering the possible interaction between the UCLA and IES-R scales [26]. All the models were adjusted for gender, age, country of residence (Italy vs. the UK), and academic performance, as these factors could influence the likelihood of the survey completion. Regression coefficients were reported, along with their 95% confidence intervals (95% CIs). Similarly, estimates for the direct, indirect, and total effects were provided. Finally, a path analysis was performed to jointly test multivariate associations among IES-R subscales (intrusion, avoidance, and hyperarousal), loneliness, and anxiety and depressive symptoms. Statistical significance was set at a *p*-value of 0.05.

## 3. Results

### 3.1. Sample Characteristics

The online survey was delivered to all the university students older than 18, gathering a total of 2979 responses. As a whole, 69% of the sample reported information on anxiety symptoms. Therefore, the final sample comprised 2055 undergraduate students from Italy and the UK, including (21.5%) men and (75.4%) women, with a mean age (standard deviation, SD) of 22.77 (5.21) years. The majority (71.2%) were in the first two years of their academic program, primarily enrolled in Psychosocial Sciences (703, 34.2%), Medical Sciences (517, 25.2%), and Applied/Natural Sciences (456, 22.2%). A total of 1298 students (63.2%) reported being on track with their studies. In open-ended responses about traumatic life experiences, the participants most frequently reported relevant health-related issues linked to the pandemic (316, 15.4%), followed by the unexpected death of a close friend or relative (82, 4.0%), domestic violence (41, 2.0%), a life-threatening illness (43, 2.1%), as well as psychological or physical abuse (22, 1.1%). PTS were identified in students with an IES-R score of ≥33 who also described a life event potentially consistent with the DSM-5 Criterion A for PTSD (408/19.9%) [1].

As compared to the students who early-quitted the survey and did not report information on anxiety symptoms, but only on sociodemographic data (N = 369), the survey completers (N = 2055) were more likely to be younger (mean age: 22.76 (SD = 5.21) vs. 23.80 (SD = 5.40); *p* < 0.001) and to reside in the UK (34.2% vs. 20.3%; *p* < 0.001). The descriptive statistics for the full sample of the survey completers are presented in Table 1, with country-specific details provided in Supplementary Table S1.

### 3.2. Clinical Assessment and Loneliness

The mean score for anxiety symptoms, as measured using the GAD-7, was 9.38 (SD = 5.32), while the average score for depressive symptoms, assessed using the PHQ-9, was 9.83 (SD = 6.21). The mean score on the IES-R was 24.85 (SD = 18.5), and the average UCLA Loneliness Scale score for the overall sample was 48.78 (SD = 12.31).

The mean scores on the assessment tools for the overall sample of students who completed the survey are reported in Table 1, with country-specific data detailed in Supplementary Table S1.

Positive, moderate-to-high correlations were observed between post-traumatic stress symptoms and both anxiety (ρ: 0.48, *p* < 0.001) and depressive symptoms (ρ: 0.48, *p* < 0.001). Similarly, loneliness was strongly associated with anxiety (ρ: 0.44, *p* < 0.001), depressive symptoms, and IES-R scores (ρ: 0.51 and 0.36, respectively; *p* < 0.001 for both), as indicated by Spearman’s correlation coefficients.

### 3.3. The Effect of Loneliness on the Relationship Between PTS and Anxiety Symptoms: Mediation Analysis

Controlling for gender, age, country, and academic performance, we assessed the joint contributions of PTS and loneliness to anxiety symptoms, exploring the relevant associations. We tested loneliness as a potential mediator of the relationship between PTS and anxiety symptoms, as assessed using GAD-7. A statistically significant direct effect of PTS on anxiety symptoms was found (coeff.: 3.73, 95% CI: 3.05; 4.45, *p* < 0.001). However, about 22% of the overall association was mediated by loneliness, as measured using the UCLA scale (indirect effect: 1.04, 95% CI: 0.59; 1.48, *p* < 0.001). These results are presented in Table 2(a).

### 3.4. The Effect of Loneliness on the Relationship Between PTS and Depressive Symptoms: Mediation Analysis

The mediating effect of loneliness on the relationship between PTS and depressive symptoms was also tested. Based on the IES-R scores, a statistically significant effect of PTS on the likelihood of higher scores on the PHQ-9 was detected (coeff.: 3.90, 95% CI: 3.27; 4.52; *p* < 0.001), with a significant contribution of loneliness to the model. Indeed, while a statistically significant direct effect of PTS on depressive symptoms was found (coeff.: 3.72, 95% CI: 3.02; 4.42, *p* < 0.001), about 33% of the overall association was mediated by loneliness, as measured using the UCLA scale (indirect effect: 1.81, 95% CI: 1.22; 2.39, *p* < 0.001). These results are presented in Table 2(b).

### 3.5. Multivariate Associations Among IES-R Subscales, Loneliness, and Anxiety and Depressive Symptoms: A Path Analysis

**Figure 1:**
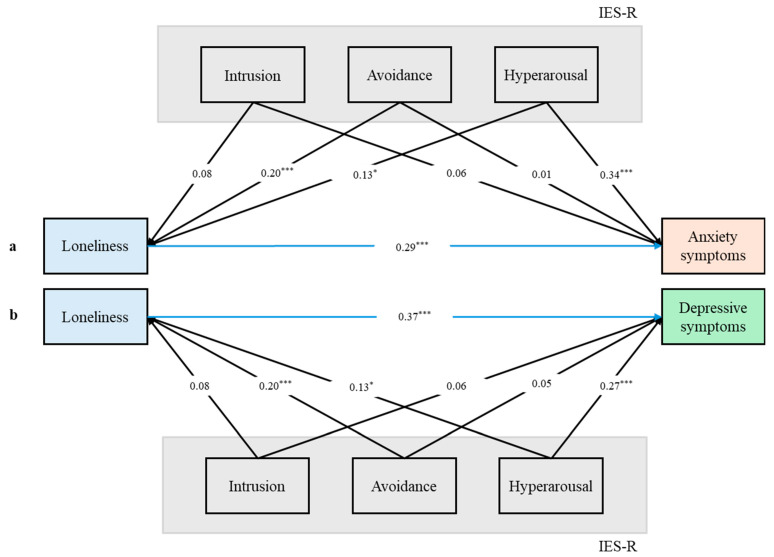
(a) Path analysis jointly testing the multivariate associations among the IES-R subscales, loneliness, and anxiety symptoms. (b) Path analysis jointly testing the multivariate associations among the IES-R subscales, loneliness, and depressive symptoms; *: *p* < 0.05; **: *p* < 0.01; ***: *p* < 0.001.

When jointly examining the multivariate associations among the three IES-R subscales, loneliness, and anxiety symptoms, significant associations were found between both the avoidance subscale (coeff.: 0.20, *p* < 0.001) and the hyperarousal subscale (coeff.: 0.13, *p* = 0.025) with loneliness. In contrast, only the hyperarousal subscale was significantly associated with anxiety symptoms (coeff.: 0.34, *p* < 0.001). Similarly, in the model exploring the associations among the IES-R subscales, loneliness, and depressive symptoms, both the avoidance (coeff.: 0.20, *p* < 0.001) and hyperarousal (coeff.: 0.13, *p* = 0.025) subscales were significantly associated with loneliness. However, only the hyperarousal subscale was significantly associated with depressive symptoms (coeff.: 0.27, *p* < 0.001). The relevant paths are displayed in Figure 1.

## 4. Discussion

To the best of our knowledge, this is the first study investigating whether loneliness can influence the relationships between PTS and both anxiety and depressive symptoms among university students. We consistently found distinct associations among PTS, loneliness, and anxiety and depressive symptoms. In addition, loneliness exerted a mediating effect on the relationships between PTS and both anxiety and depressive symptoms. Finally, significant associations between the IES-R’s hyperarousal subscale and both anxiety and depressive symptoms were found.

Our findings are consistent with those in prior research supporting an association between PTS and anxiety symptoms [8]. This relationship may be partially explained by overlapping clinical features, including avoidance behaviors, sleep disturbances, and hypervigilance [9]. The high rates of comorbidity between PTS and anxiety disorders further highlight the likelihood of shared pathophysiological mechanisms [27]. Specifically, the dysregulation of the stress–response system, notably involving the hypothalamus–pituitary–adrenal gland (HPA) axis and the autonomic nervous system, has been proposed as a common neurobiological substrate contributing to the development and persistence of both PTS and anxiety-related symptoms [27,28]. Moreover, the association between PTS and depressive symptoms can likely reflect the impacts of traumatic experiences as a shared etiological factor underpinning both post-traumatic and depressive psychopathologies [9]. Once again, this relationship may be reinforced by overlapping clinical characteristics, namely, negative changes in cognition and mood [9,29].

While growing evidence supports a bidirectional relationship between loneliness and depression over time [16,17,18], anxiety has also been shown to predict future experiences of loneliness across various age groups, indicating a reciprocal dynamic [13,14,15]. On the one hand, loneliness has been identified as a mediator in the relationship between anxiety and depressive symptoms and is strongly associated with social anxiety in both younger and older populations [30]: shared features, including social withdrawal, negative social cognition, impaired interpersonal functioning, and reduced quality of social interactions, may contribute to and exacerbate the experience of loneliness [13]. As a matter of fact, loneliness is a complex and multidimensional construct, encompassing both the quality of relationships with one’s family of origin and the sense of belonging to a broader social network [31,32]. As such, the relationship between social interaction and perceived loneliness is not always straightforward [33].

Interestingly, our findings also reveal a mediating role of loneliness in the relationships between PTS and both anxiety and depressive symptoms. This is consistent with recent research suggesting that feelings of loneliness can influence the relationship between social support and individuals’ abilities to cope with trauma and stress, thereby playing a relevant role in psychological adaptation [34,35]. Indeed, theoretical frameworks differentiate two key dimensions of loneliness: not only emotional loneliness, defined by the absence of a close attachment figure, but also social loneliness, characterized by the lack of a broader social network that fosters social support and connection [36]. While positive social interactions, supportive relationships, and a strong sense of belonging may serve as protective factors against the detrimental effects of overwhelming stress and trauma, perceived loneliness could exacerbate the negative consequences of traumatic events [11,12]. Furthermore, the interplay between PTS and loneliness may possibly follow different paths. Whereas research suggests that individuals with PTS are more likely to experience loneliness, possibly due to trauma-associated social withdrawal [37,38], loneliness, as such, also seems to predict later PTS [37]. Other factors, such as negative thinking patterns, sleep disturbances, and relational difficulties, may also contribute to this mutual influence [39]. Loneliness itself can be perpetuated by negative cognitive biases, like heightened vigilance for social threats [40], a trait also found in subjects suffering from PTS who often feel persistently threatened, even without external triggers [41]. While loneliness is associated with symptoms often reported by trauma-exposed individuals, including intrusions and avoidance [42,43], its typical features, like feelings of alienation and detachment, may conceptually overlap with symptoms of post-traumatic stress, potentially complicating the distinction of the two conditions [42,44].

Finally, significant associations between the IES-R’s hyperarousal subscale and both anxiety and depressive symptoms were uncovered by path analysis. Hyperarousal refers to a state of psychological activation that reflects an excessive and persistent stress response [1]. It includes symptoms such as an increased startle response, irritability, poor sleep, hypervigilance, and impaired concentration [45]. This heightened arousal perpetuates threat sensitivity and interferes with emotional regulation, contributing to the persistence of PTS [45]. Beyond this, hyperarousal is also closely associated with anxiety symptoms, due to overlapping features, such as restlessness and excessive alertness [46]. Individuals experiencing hyperarousal often report anticipatory anxiety and difficulty tolerating uncertainty, which reinforce anxious cognition and behavior [47]. While hyperarousal is more classically linked to anxiety, it can also contribute to depressive symptoms [48,49]. Indeed, chronic sleep disturbances, emotional exhaustion, and sustained stress reactivity associated with hyperarousal can lead to anhedonia, irritability, and cognitive fatigue, which represent core elements of depression [49]. In addition, prolonged hyperarousal may deplete coping resources, thus increasing vulnerability to depressive episodes [50,51]. This finding seems to underscore the role of hyperarousal in the broader emotional dysregulation that follows trauma exposure and supports its potential utility as a transdiagnostic indicator of affective symptoms in trauma-related psychopathology [45].

Considering the influences of cultural and lifestyle differences among students is essential for interpreting our findings. In the UK, students often live on campus or in shared accommodations, which can facilitate peer interaction and a stronger sense of belonging, possibly reducing the effects of stressful events [52,53]. In addition, our results suggest that campus life may also enhance social experiences, potentially fostering academic engagement. This is reflected in the higher proportion of UK students who reported being on track with their exams [53,54]. However, this setting may also introduce new responsibilities, increased autonomy, and reduced family contact, factors that have been associated with greater psychological distress and higher rates of depressive symptoms [55,56]. In contrast, the majority of the Italian students typically reside with their families, a living arrangement that may limit opportunities for peer bonding and increase the risks of social isolation and anxiety [57,58].

Given the interplay among loneliness, PTS, and anxiety and depressive symptoms, interventions addressing loneliness and social isolation should be prioritized as a strategy to promote psychological well-being [59]. This is particularly relevant for individuals exposed to traumatic experiences [14,60]. For these students, the absence of a reliable and emotionally supportive social network can amplify the psychological impacts of trauma and stress, leading to poor mental health [14,60]. Promoting environments that facilitate meaningful, face-to-face interactions, such as group-based activities, peer-support programs, and community-building initiatives, could, therefore, significantly reduce psychological and mental distress among university students [61,62,63].

Our findings should be interpreted with caution, considering some methodological limitations. First, the cross-sectional nature of the study does not allow us to draw conclusions about causal relationships among PTS, the dependent variables, and the mediating factor.

While we observed significant associations, the directionality of these relationships cannot be determined. It is plausible, based on existing theoretical frameworks and prior longitudinal research, that anxiety, depressive symptoms, and PTS can contribute to increased feelings of loneliness; however, the reverse, namely, that loneliness may exacerbate and potentially mediate psychological distress, is also theoretically and empirically supported [31,36].

Second, the use of an open-ended survey question limited our ability to fully assess the depth, context, and diagnostic relevance of reported traumatic events. We used a cutoff score of ≥33 on the IES-R scale to quantify post-traumatic stress symptoms, even though we were not always able to determine whether the reported traumatic events fully met the DSM-5 Criterion A for PTSD. Future studies should incorporate more refined measures to provide a richer understanding of how these events could more specifically influence mental health outcomes.

Third, the timing of the survey, administered shortly after the main wave of the COVID-19 pandemic, likely contributed to the high percentage of students reporting traumatic events, many of which may reflect general pandemic-related stress rather than events that meet the specific criterion outlined in the DSM-5 for trauma [64]. Moreover, since clinical domains were measured using brief screening scales, newly designed research would benefit from a more comprehensive and structured diagnostic reporting of clinical conditions in order to reduce recall bias and/or under-reporting, as well as potential misclassification. Furthermore, our methodology, reliant on an online survey for clinical data collection, inherently presents various limitations, notably concerning sampling issues [65]. Similarly, the selected participants may not entirely reflect the broader university student population. They are likely to be individuals already interested in mental health issues, potentially involving a selection bias. However, the survey was conducted within a private household setting, which has been shown to mitigate under-reporting and bias, facilitated by perceived privacy and anonymity, thereby addressing potential stigmatization or embarrassment.

## 5. Conclusions

Our findings support the well-established associations between PTS and anxiety as well as depressive symptoms. In addition, the key mediating role of loneliness in both associations was highlighted. Therefore, students with PTS who report anxiety or depressive symptoms might also benefit from the assessment of feelings of loneliness. Addressing the specific treatment needs of vulnerable university students exposed to traumatic events, particularly through targeted interventions aimed at reducing loneliness, may ultimately lead to improved mental health outcomes.

## Figures and Tables

**Table 1:** Sociodemographic and clinical characteristics and differences between individuals with and without PTS.

Characteristics	Overall Sample	PTS **		*p*-Value *
	N = 2055	No PTS (N = 944)	PTS (N = 408)	
	*N (%) or Mean (SD)*	*N (%) or Mean (SD)*	*N (%) or Mean (SD)*	
*Gender*				
Women	1550 (75.4%)	722 (76.5%)	317 (77.7%)	*p* = 0.25
Men	442 (21.5%)	193 (20.4%)	71 (17.4%)	
*Age, years*	22.77 (5.21)	23.12 (5.54)	22.76 (4.50)	*p* = 0.10
*Years of Study*				
First	664 (32.3%)	294 (31.1%)	159 (39.0%)	***p* = 0.012**
Second	634 (38.9%)	312 (33.1%)	101 (24.8%)	
Third	445 (21.7%)	220 (23.3%)	89 (21.8%)	
Fourth	119 (5.8%)	51 (5.4%)	27 (6.6%)	
Fifth–Sixth	126 (6.1%)	63 (6.7%)	29 (7.1%)	
***Degree Program Area***				
Applied/Natural Sciences ^1^	456 (22.2%)	215 (22.8%)	92 (22.6%)	***p* = 0.002**
Economic/Legal Sciences ^2^	343 (16.7%)	139 (14.7%)	56 (13.7%)	
Medical Sciences ^3^	517 (25.2%)	251 (26.6%)	86 (21.1%)	
Psychosocial Sciences ^4^	703 (34.2%)	319 (33.8%)	166 (40.7%)	
***International Student***	103 (5.0%)	38 (4.0%)	30 (7.4%)	***p* = 0.014**
***Out-of-Town Student***	686 (33.4%)	315 (33.4%)	148 (36.3%)	*p* = 0.29
***Employment***				
Non-worker	683 (33.2%)	302 (32.0%)	137 (33.6%)	*p* = 0.51
Part-time or full-time worker	1332 (64.8%)	625 (66.2%)	261 (64.0%)	
***Marital Status***				
Single	1047 (51.0%)	475 (50.3%)	189 (46.3%)	*p* = 0.26
In a committed relationship	945 (46.0%)	441 (46.7%)	201 (49.3%)	
***Accommodation***				
With family of origin	1071 (52.1%)	494 (52.3%)	203 (49.8%)	***p* < 0.001**
On campus	302 (14.7%)	126 (13.4%)	60 (14.7%)	
With roommates outside of campus	375 (18.3%)	184 (19.5%)	70 (17.2%)	
With partner	164 (8.0%)	77 (8.2%)	34 (8.3%)	
Alone	103 (5.0%)	46 (4.9%)	29 (7.1%)	
***On Track with Exams***	1298 (63.2%)	629 (66.6%)	229 (56.1%)	***p* = 0.002**
***Social Interactions—family***				
Daily/more times per week	1410 (68.6%)	649 (68.8%)	275 (67.4%)	*p* = 0.83
Weekly	251 (12.2%)	123 (13.0%)	49 (12.0%)	
Monthly or less	349 (17.0%)	156 (16.5%)	71 (17.4%)	
***Social Interactions—partner***				
Daily/more times per week	616 (30.0%)	284 (30.1%)	128 (31.4%)	*p* = 0.46
Weekly	152 (7.4%)	78 (8.3%)	27 (6.6%)	
Monthly or less	287 (14.0%)	130 (13.8%)	63 (15.4%)	
***Social Interactions—friends***				
Daily/more times per week	646 (31.4%)	291 (30.8%)	130 (31.9%)	*p* = 0.56
Weekly	689 (33.5%)	331 (35.1%)	149 (36.5%)	
Monthly or less	693 (33.7%)	315 (33.4%)	123 (30.2%)	
***Social Interactions—classmates***				
Daily/more times per week	901 (43.8%)	421 (44.6%)	179 (43.8%)	*p* = 0.70
Weekly	233 (11.3%)	106 (11.2%)	51 (12.5%)	
Monthly or less	801 (39.0%)	365 (38.7%)	149 (36.5%)	
***Anxiety Symptoms (GAD-7)***	9.38 (5.32)	8.31 (4.52)	13.18 (5.13)	***p* < 0.001**
***Depressive Symptoms (PHQ-9)***	9.83 (6.21)	8.45 (5.32)	14.09 (6.18)	***p* < 0.001**
***Loneliness (UCLA)***	48.78 (12.31)	46.80 (11.91)	54.43 (10.65)	***p* < 0.001**

* Statistically significant differences between groups defined by the Impact of Event Scale—Revised (IES-R) cutoff score (*p* < 0.05). ** PTS: defined as an IES-R score of ≥33 combined with a life event potentially fulfilling DSM-5 Criterion A for PTSD. Data for the Impact of Event Scale—Revised (IES-R) were available for 1352 (65.79%) participants. Missing values are not reported in the table. Bold formatting was used to denote *p*-values below the 0.05 threshold for statistical significance. SD: Standard deviation; PTS: post-traumatic stress symptoms. ^1^ Applied/Natural Sciences: Biochemistry; Biology; Biomedical Engineering; Biomedical Sciences; Computer Sciences; Geology; Math. ^2^ Economic/Legal Sciences: Economics; Hospitality and Tourism; Law; Politics. ^3^ Medical Sciences: Medicine and Surgery; Nursing; Nutrition; Paramedicine; Veterinary Medicine. ^4^ Psychosocial Sciences: Education Sciences; Intercultural Communication; Psychology; Sociology.

**Table 2:** (**a**) Test of mediation between PTS and anxiety symptoms (GAD-7): Loneliness was assessed using the UCLA Loneliness Scale (Version 3) *. (**b**) Test of mediation between PTS and depressive symptoms (PHQ-9): Loneliness was assessed using the UCLA Loneliness Scale (Version 3) *.

**2(a) GAD-7**	**Coefficient**	**Standard Error**	**95% CI**	**β ^a^**	***p*-Value**
Loneliness	0.14	0.01	0.12; 0.16	0.33	<0.001
PTS	3.66	0.28	3.12; 4.22	0.32	<0.001
*Test of mediation*					
Indirect effect	1.04	0.23	0.59; 1.48		<0.001
Direct effect	3.73	0.35	3.05; 4.45		<0.001
Total effect	4.77	0.32	4.15; 5.40		<0.001
**2(b) PHQ-9**	**Coefficient**	**Standard Error**	**95% CI**	**β ^b^**	***p*-Value**
Loneliness	0.21	0.01	0.19; 0.23	0.42	<0.001
PTS	3.90	0.32	3.27; 4.52	0.29	<0.001
*Test of mediation*					
Indirect effect	1.81	0.30	1.22; 2.39		<0.001
Direct effect	3.72	0.36	3.02; 4.42		<0.001
Total effect	5.53	0.38	6.79; 6.27		<0.001

N = 1099, * controlling for gender, age, country (Italy vs. the UK), and academic performance. PTS: post-traumatic stress symptoms; β ^a^: beta coefficient and the related estimates for the association of the selected independent variable with anxiety symptoms; β ^b^: beta coefficient and the related estimates for the association of the selected independent variable with depressive symptoms.

## Data Availability

The raw data supporting the conclusions of this article will be made available by the authors on request due to ethical/legal requirements.

## Supplementary Materials

The following supporting information can be downloaded at https://www.mdpi.com/article/10.3390/brainsci15080787/s1: Table S1: Sample characteristics by country.

## Abbreviations

The following abbreviations are used in this manuscript:

PTS	Post-traumatic stress symptoms
Unimib	University of Milano–Bicocca
UoS	University of Surrey
GAD-7	Generalized Anxiety Disorder Scale-7
PHQ-9	Patient Health Questionnaire-9
IES-R	Impact of Event Scale—Revised
UCLA	UCLA Loneliness Scale (Version 3)

## References

[1] Psychiatric Association (2022). Diagnostic and Statistical Manual of Mental Disorders.

[2] Tibubos A.N., Burghardt J., Klein E.M., Brähler E., Jünger C., Michal M., Wiltink J., Wild P.S., Münzel T., Singer S. (2021). Frequency of stressful life events and associations with mental health and general subjective health in the general population. J. Public Health.

[3] Kring L., Iversen E., Ibsen B., Fehsenfeld M. (2024). Exploring the impact of stressful life events on quality of life: Meaning making and narrative reconstruction. Int. J. Qual. Stud. Health Well-Being.

[4] Roberts A.L., Dohrenwend B.P., Aiello A., Wright R.J., Galea S., Maercker A., Galea S., Koenen K.C. (2012). The stressor criterion for posttraumatic stress disorder: Does it matter?. J. Clin. Psychiatry.

[5] Kira I.A., Fawzi M.H., Shuwiekh H., Lewandowski L., Ashby J.S., Ibraheem B.A. (2019). Do adding attachment, oppression, cumulative and proliferation trauma dynamics to PTSD criterion ‘A’ improve its predictive validity: Toward a paradigm shift?. Curr. Psychol..

[6] Briggs A., Clark J., Hall I. (2012). Building bridges: Understanding student transition to university. Qual. High. Educ..

[7] Pedrelli P., Nyer M., Yeung A., Zulauf C., Wilens T. (2015). College Students: Mental Health Problems and Treatment Considerations. Acad. Psychiatry.

[8] Williamson J.B., Jaffee M.S., Jorge R.E. (2021). Posttraumatic Stress Disorder and Anxiety-Related Conditions. Continuum.

[9] Bryant R.A. (2019). Post-traumatic stress disorder: A state-of-the-art review of evidence and challenges. World Psychiatry.

[10] Sheldon E., Simmonds-Buckley M., Bone C., Mascarenhas T., Chan N., Wincott M., Gleeson H., Sow K., Hind D., Barkham M. (2021). Prevalence and risk factors for mental health problems in university undergraduate students: A systematic review with meta-analysis. J. Affect. Disord..

[11] Acoba E.F. (2024). Social support and mental health: The mediating role of perceived stress. Front. Psychol..

[12] Weziak-Bialowolska D., Bialowolski P., Lee M.T., Chen Y., VanderWeele T.J., McNeely E. (2022). Prospective associations between social connectedness and mental health. Evidence from a longitudinal survey and health insurance claims data. Int. J. Public Health.

[13] Danneel S., Maes M., Vanhalst J., Bijttebier P., Goossens L. (2018). Developmental change in loneliness and attitudes toward aloneness in adolescence. J. Youth Adolesc..

[14] Richardson T., Elliott P., Roberts J., Jansen M. (2017). Longitudinal relationship between loneliness and mental health in university students. J. Public Ment. Health.

[15] Riboldi I., Capogrosso C.A., Piacenti S., Calabrese A., Lucini Paioni S., Bartoli F., Crocamo C., Carrà G., Armes J., Taylor C. (2023). Mental health and COVID-19 in university students: Findings from a qualitative, comparative study in Italy and the UK. Int. J. Environ. Res. Public Health.

[16] Mann F., Wang J., Pearce E., Ma R., Schlief M., Lloyd-Evans B., Ikhtabi S., Johnson S. (2022). Loneliness and the onset of new mental health problems in the general population. Soc. Psychiatry Psychiatr. Epidemiol..

[17] Achterbergh L., Pitman A., Birken M., Pearce E., Sno H., Johnson S. (2020). The experience of loneliness among young people with depression: A qualitative meta-synthesis of the literature. BMC Psychiatry.

[18] Riboldi I., Crocamo C., Piacenti S., Capogrosso C.A., Calabrese A., Lucini Paioni S., Bartoli F., Armes J., Taylor C., Carrà G. (2025). Mental health and loneliness in university students: A structural equation modelling comparing Italy and the UK. Int. J. Soc. Psychiatry.

[19] von Elm E., Altman D.G., Egger M., Pocock S.J., Gøtzsche P.C., Vandenbroucke J.P., STROBE Initiative (2008). The Strengthening the Reporting of Observational Studies in Epidemiology (STROBE) statement: Guidelines for reporting observational studies. J. Clin. Epidemiol..

[20] Spitzer R.L., Kroenke K., Williams J.B., Löwe B. (2006). A brief measure for assessing generalized anxiety disorder: The GAD-7. Arch. Intern. Med..

[21] Kroenke K., Spitzer R.L., Williams J.B. (2001). The PHQ-9: Validity of a brief depression severity measure. J. Gen. Intern. Med..

[22] Spitzer R.L., Kroenke K., Williams J.B. (1999). Validation and utility of a self-report version of PRIME-MD: The PHQ primary care study. JAMA.

[23] Weiss D.S., Marmar C.R., Wilson J.P., Keane T.M. (1997). The Impact of Event Scale-Revised. Assessing Psychological Trauma and PTSD.

[24] Russell D.W. (1996). UCLA Loneliness Scale (Version 3): Reliability, validity, and factor structure. J. Pers. Assess..

[25] Kenny D.A. Mediation. Online Resource 2021. http://davidakenny.net/cm/mediate.htm.

[26] Narita Z.C., Miyashita M., Furukawa T.A., Nishida A. (2025). Key considerations in mediation analysis for psychiatric research. JAMA Psychiatry.

[27] Chiu H.T.S., Low D.C.W., Chan A.H.T., Meiser-Stedman R. (2024). Relationship between anxiety sensitivity and post-traumatic stress symptoms in trauma-exposed adults: A meta-analysis. J. Anxiety Disord..

[28] Kinlein S.A., Karatsoreos I.N. (2020). The hypothalamic-pituitary-adrenal axis as a substrate for stress resilience: Interactions with the circadian clock. Front. Neuroendocrinol..

[29] Miethe S., Wigger J., Wartemann A., Trautmann S. (2023). Posttraumatic stress symptoms and its association with rumination, thought suppression and experiential avoidance: A systematic review and meta-analysis. J. Psychopathol. Behav. Assess..

[30] Teo A.R., Lerrigo R., Rogers M.A. (2013). The role of social isolation in social anxiety disorder: A systematic review and meta-analysis. J. Anxiety Disord..

[31] Wang J., Mann F., Lloyd-Evans B., Ma R., Johnson S. (2018). Associations between loneliness and perceived social support and outcomes of mental health problems: A systematic review. BMC Psychiatry..

[32] Yanguas J., Pinazo-Henandis S., Tarazona-Santabalbina F.J. (2018). The complexity of loneliness. Acta Biomed..

[33] Qualter P., Vanhalst J., Harris R., Van Roekel E., Lodder G., Bangee M., Maes M., Verhagen M. (2015). Loneliness across the life span. Perspect. Psychol. Sci..

[34] Calhoun C.D., Stone K.J., Cobb A.R., Patterson M.W., Danielson C.K., Bendezú J.J. (2022). The role of social support in coping with psychological trauma: An integrated biopsychosocial model for posttraumatic stress recovery. Psychiatr. Q..

[35] O’Riordan A., Costello A.M. (2025). Loneliness mediates the association between trait social anxiety and cardiovascular reactivity to acute psychological stress. Int. J. Psychophysiol..

[36] Pitman A., Mann F., Johnson S. (2018). Advancing our understanding of loneliness and mental health problems in young people. Lancet Psychiatry.

[37] Thompson N.J., Fiorillo D., Rothbaum B.O., Ressler K.J., Michopoulos V. (2018). Coping strategies as mediators in relation to resilience and posttraumatic stress disorder. J. Affect. Disord..

[38] van der Velden P.G., Pijnappel B., van der Meulen E. (2018). Potentially traumatic events have negative and positive effects on loneliness, depending on PTSD-symptom levels: Evidence from a population-based prospective comparative study. Soc. Psychiatry Psychiatr. Epidemiol..

[39] Fox R., McHugh Power J., Coogan A.N., Beekman A.T.F., van Tilburg T.G., Hyland P. (2021). Posttraumatic stress disorder and loneliness are associated over time: A longitudinal study on PTSD symptoms and loneliness among older adults. Psychiatry Res..

[40] Hawkley L.C., Cacioppo J.T. (2010). Loneliness matters: A theoretical and empirical review of consequences and mechanisms. Ann. Behav. Med..

[41] Williamson J.B., Porges E.C., Lamb D.G., Porges S.W. (2015). Maladaptive autonomic regulation in PTSD accelerates physiological aging. Front. Psychol..

[42] Dagan Y., Yager J. (2019). Addressing loneliness in complex PTSD. J. Nerv. Ment. Dis..

[43] Matthews T., Danese A., Gregory A.M., Caspi A., Moffitt T.E., Arseneault L. (2017). Sleeping with one eye open: Loneliness and sleep quality in young adults. Psychol. Med..

[44] Palmer B.W., Hussain M.A., Lohr J.B. (2022). Loneliness in posttraumatic stress disorder: A neglected factor in accelerated aging?. J. Ageing Longev..

[45] Ressler K.J., Berretta S., Bolshakov V.Y., Rosso I.M., Meloni E.G., Rauch S.L., Carlezon W.A. (2022). Post-traumatic stress disorder: Clinical and translational neuroscience from cells to circuits. Nat. Rev. Neurol..

[46] Sherin J.E., Nemeroff C.B. (2011). Post-traumatic stress disorder: The neurobiological impact of psychological trauma. Dialogues Clin. Neurosci..

[47] Brown V.M., Price R., Dombrovski A.Y. (2023). Anxiety as a disorder of uncertainty: Implications for understanding maladaptive anxiety, anxious avoidance, and exposure therapy. Cogn. Affect. Behav. Neurosci..

[48] Cui L., Li S., Wang S., Wu X., Liu Y., Yu W., Wang Y., Tang Y., Xia M., Li B. (2024). Major depressive disorder: Hypothesis, mechanism, prevention and treatment. Signal Transduct. Target Ther..

[49] Ryan E., Hore K., Power J., Jackson T. (2023). The relationship between physician burnout and depression, anxiety, suicidality and substance abuse: A mixed methods systematic review. Front. Public Health.

[50] Pérez L.G., Abrams M.P., López-Martínez A.E., Asmundson G.J. (2012). Trauma exposure and health: The role of depressive and hyperarousal symptoms. J. Trauma Stress.

[51] Brewin C.R., Atwoli L., Bisson J.I., Galea S., Koenen K., Lewis-Fernández R. (2025). Post-traumatic stress disorder: Evolving conceptualization and evidence, and future research directions. World Psychiatry.

[52] European Commission EUROSTAT. Learning mobility statistics. Online Resource 2023. https://ec.europa.eu/eurostat/statistics-explained/index.php?title=Learning_mobility_statistics.

[53] Holliman A.J., Waldeck D., Jay B., Murphy S., Atkinson E., Collie R.J., Martin A. (2021). Adaptability and social support: Examining links with psychological wellbeing among UK students and non-students. Front. Psychol..

[54] Boulton C.A., Hughes E., Kent C., Smith J.R., Williams H.T.P. (2019). Student engagement and wellbeing over time at a higher education institution. PLoS ONE.

[55] Worsley J.D., Harrison P., Corcoran R. (2021). The role of accommodation environments in student mental health and wellbeing. BMC Public Health.

[56] Hood C.O., Thomson Ross L., Wills N. (2020). Family factors and depressive symptoms among college students: Understanding the role of self-compassion. J. Am. Coll. Health.

[57] European Commission (2020). EUROSTAT. When Are They Ready to Leave the Nest? Online Resource. https://ec.europa.eu/eurostat/web/products-eurostat-news/-/edn-20200812-1.

[58] Calandri E., Graziano F., Rollé L. (2021). Social media, depressive symptoms and well-being in early adolescence: The moderating role of emotional self-efficacy and gender. Front. Psychol..

[59] Ellard O.B., Dennison C., Tuomainen H. (2023). Interventions addressing loneliness amongst university students: A systematic review. Child Adolesc. Ment. Health.

[60] Brandt L., Liu S., Heim C., Heinz A. (2022). The effects of social isolation stress and discrimination on mental health. Transl. Psychiatry.

[61] Gold J.A., Bentzley J.P., Franciscus A.M., Forte C., De Golia S.G. (2019). An intervention in social connection: Medical student reflection groups. Acad. Psychiatry.

[62] Eccles A.M., Qualter P. (2021). Alleviating loneliness in young people—A meta-analysis of interventions. Child Adolesc. Ment. Health.

[63] Osborn T., Weatherburn P., French R.S. (2021). Interventions to address loneliness and social isolation in young people: A systematic review of the evidence on acceptability and effectiveness. J. Adolesc..

[64] Norrholm S.D., Zalta A., Zoellner L., Powers A., Tull M.T., Reist C., Schnurr P.P., Weathers F., Friedman M.J. (2021). Does COVID-19 count?: Defining Criterion A trauma for diagnosing PTSD during a global crisis. Depress. Anxiety.

[65] Andrade C. (2020). The limitations of online surveys. Indian J. Psychol. Med..

