# Supplementary material for: Understanding the Role of Loneliness in the Relationships Between Post-Traumatic Stress Symptoms and Both Anxiety and Depressive Symptoms Among University Students: A Mediation Analysis
Source: Brain Sci. 2025 Jul 24;15(8):787. doi: 10.3390/brainsci15080787 (PMC12384088; doi:10.3390/brainsci15080787)
Supplement: Supplementary file 1 [file brainsci-15-00787-s001.zip › brainsci-3714007-supplementary.pdf]

**Supplementary Table S1**. Sample characteristics by country.

| Characteristics | Italy<br>N=1352<br>*N (%) or Mean (SD)* | UK<br>N=703<br>*N (%) or Mean (SD)* |
|---|---|---|
| ***Gender*** | | |
| Women | 1039 (76.8%) | 511 (72.7%) |
| Men | 281 (20.8%) | 161 (22.9%) |
| ***Age**, yrs.* | 23.34 (4.98) | 21.70 (5.46) |
| ***Years of study*** | | |
| First | 402 (29.7%) | 262 (37.3%) |
| Second | 419 (31.0%) | 215 (30.6%) |
| Third | 298 (22.0%) | 147 (20.9%) |
| Fourth | 63 (4.7%) | 55 (7.8%) |
| Fifth - Sixth | 115 (8.5%) | 11 (1.6%) |
| ***Degree Programme Area*** | | |
| Applied/Naturale Sciences[1] | 345 (25.5%) | 111(15.8%) |
| Economic/Legal Sciences[2] | 239 (17.7%) | 104 (14.8%) |
| Medical Sciences[3] | 139 (10.3%) | 378 (53.8%) |
| Psychosocial Sciences[4] | 628 (46.4%) | 75 (10.7%) |
| ***International student*** | 19 (1.4%) | 84 (12.0%) |
| ***Out-of-town student*** | 413 (30.6%) | 273 (38.8%) |
| ***Employment*** | | |
| Non-worker | 665 (49.2%) | 18 (2.6%) |
| Part-time or full-time worker | 658 (48.7%) | 674 (95.9%) |
| ***Accommodation*** | | |
| With family of origin | 974 (72.0%) | 97 (13.8%) |
| With roommates | 167 (12.4%) | 208 (29.6%) |
| With partner | 28 (2.1%) | 274 (39.0%) |
| Alone | 62 (4.6%) | 41 (5.8%) |
| ***On track with exams*** | 771 (57.0%) | 527 (75.0%) |
| ***Social interactions – family*** | | |
| Daily/more time a week | 1045 (77.3%) | 365 (51.9%) |
| Weekly | 117 (8.7%) | 134 (19.1%) |
| Monthly or less | 168 (12.4%) | 181 (25.8%) |
| ***Social interactions – partner*** | | |
| Daily/more time a week | 550 (40.7%) | 67 (9.4%) |
| Weekly | 97 (7.2%) | 55 (7.8%) |
| Monthly or less | 68 (5.0%) | 219 (31.2%) |
| ***Social interactions – friends*** | | |
| Daily/more time a week | 518 (38.3%) | 128 (18.2%) |
| Weekly | 585 (43.3%) | 104 (14.8%) |
| Monthly or less | 227 (16.8%) | 466 (66.3%) |
| ***Social interactions – classmates*** | | |
| Daily/more time a week | 768 (56.8%) | 133 (18.9%) |
| Weekly | 149 (11.0%) | 84 (12.0%) |
| Monthly or less | 338 (25.0%) | 463 (65.9%) |
| ***Anxiety symptoms (GAD-7)*** | 9.59 (4.96) | 8.86 (5.91) |
| ***Depressive symptoms (PHQ-9)*** | 9.34 (5.95) | 10.81 (6.56) |
| ***Loneliness (UCLA)*** | 49.02 (12.20) | 48.30 (12.52) |

| | | |
|---|---|---|
| ***PTS (IES-R)*** | 25.56 (17.45) | 23.45 (19.10) |

Missing values are not reported in the table.

SD: Standard deviation; PTS: post-traumatic stress symptoms

[1]Applied/Natural Sciences: Biochemistry; Biology; Biomedical Engineering; Biomedical Sciences; Computer Sciences; Geology; Maths.

[2]Economic/Legal Sciences: Economics; Hospitality and Tourism; Law; Politics.

[3]Medical sciences: Medicine and Surgery; Nursing; Nutrition; Paramedics; Veterinary Medicine.

[4]Psychosocial sciences: Education Sciences; Intercultural communication; Psychology; Sociology.
